# The Effect of Acute Knee Injuries and Related Knee Surgery on Serum Levels of Pro- and Anti-inflammatory Lipid Mediators and Their Associations With Knee Symptoms

**DOI:** 10.1177/03635465241228209

**Published:** 2024-02-26

**Authors:** James Turnbull, Rakesh R. Jha, David A. Barrett, Ana M. Valdes, Jennifer Alderson, Andrew Williams, Tonia L. Vincent, Fiona E. Watt, Victoria Chapman

**Affiliations:** * NIHR Nottingham Biomedical Research Centre, University of Nottingham, Nottingham, UK; † Centre for Analytical Bioscience, Advanced Materials and Healthcare Technologies Division, School of Pharmacy, University of Nottingham, Nottingham, UK; ‡ School of Life Sciences, University of Nottingham, Nottingham, UK; § Injury, Recovery, and Inflammation Sciences, School of Medicine, University of Nottingham, Nottingham, UK; ‖ Centre for Osteoarthritis Pathogenesis Versus Arthritis, Kennedy Institute of Rheumatology, University of Oxford, Oxford, UK; ¶ Fortius Clinic, London, UK; # Department of Immunology and Inflammation, Imperial College London, London, UK; Investigation performed at the University of Nottingham, Nottingham, UK and the University of Oxford, Oxford, UK

**Keywords:** knee injury, inflammation, bioactive lipids, longitudinal, osteoarthritis, pathogenesis, biomarker

## Abstract

**Background:**

Despite an acute knee injury being a major risk factor for osteoarthritis, the factors that initiate and maintain this risk of longer-term knee symptoms are poorly understood. Bioactive lipids derived from omega-3 and -6 polyunsaturated fatty acids have key roles in the regulation of the inflammatory response and have been linked to joint damage and osteoarthritis pain in translational models.

**Hypothesis:**

There would be associations between systemic levels of bioactive lipids and knee symptoms longitudinally after an acute knee injury and related knee surgery.

**Study Design:**

Controlled laboratory study.

**Methods:**

This study analyzed a subset of young, active adults who had sustained an acute knee injury (recruited via a surgical care pathway) and healthy age- and sex-matched controls. Surgery, if performed, was conducted after the baseline serum sample was taken and before the 3-month and 2-year visits. Liquid chromatography–tandem mass spectrometry of 41 bioactive lipids was carried out in sera of (1) 47 injured participants (median age, 28 years) collected at baseline (median, 24 days after injury), 3 months, and 2 years, along with the Knee injury and Osteoarthritis Outcome Score, and (2) age- and sex-matched controls.

**Results:**

Levels of the omega-3 polyunsaturated fatty acids eicosapentaenoic acid (*P*≤ .0001) and docosahexaenoic acid (*P*≤ .0001) and the pro-resolving lipid mediators 17– and 14–hydroxydocosahexaenoic acid, and 18-hydroxyeicosapentaenoic acid were all significantly greater at baseline in injured participants compared with the later time points and also higher than in healthy controls (*P* = .0019 and *P*≤ .0001, respectively). Levels of pro-inflammatory prostaglandins E2 and D2, leukotriene B4, and thromboxane B2 were significantly lower at baseline compared with the later time points. Higher levels of 8,9–, 11,12–, and 14,15–dihydroxyeicosatrienoic acid (DHET) were cross-sectionally associated with more severe knee pain/symptoms according to the Knee injury and Osteoarthritis Outcome Score at 2 years (*P* = .0004, *R*^2^ = 0.251; *P* = .0002, *R*^2^ = 0.278; and *P* = .0012, *R*^2^ = 0.214, respectively).

**Conclusion:**

The profile of pro-resolving versus pro-inflammatory lipids at baseline suggests an initial activation of pro-resolution pathways, followed by the later activation of pro-inflammatory pathways.

**Clinical Relevance:**

In this largely surgically managed cohort, the association of soluble epoxide hydrolase metabolites, the DHETs, with more severe knee symptoms at 2 years provides a rationale for further investigation into the role of this pathway in persisting knee symptoms in this population, including potential therapeutic strategies.

Acute knee injuries, including anterior cruciate ligament ruptures, meniscal tears, and intra-articular fractures,^14^ can lead to chronic knee pain and are a major risk factor for osteoarthritis,^13,23^ with most injury types being associated with a 4- to 9-fold increase in the risk.^26^ Typically, mechanistic knee pain studies focus on events at the time of the diagnosis of the established joint disease (ie, knee osteoarthritis), which often coincides with older age and higher rates of comorbidities.^19^ Studies looking at early structural posttraumatic osteoarthritis are challenging, as changes observed on imaging can often overlap with those of the injury, and there is little consensus on what constitutes early and posttraumatic osteoarthritis.^12^ The molecular events leading up to chronic knee pain associated with knee osteoarthritis are less well understood.

Bioactive lipids have fundamental roles in the initiation, maintenance, and resolution of inflammation as well as the progression to pathological damage.^6^ At the time of an injury, there is an immediate inflammatory response in the joint, and it has been reported that symptoms at the time of a knee injury are associated with higher synovial fluid levels of interleukin-6, monocyte chemoattractant protein 1 (MCP-1; also known as CCL-2), matrix metalloproteinase 3, tissue inhibitor of metalloproteinases 1, activin A, and tumor necrosis factor–stimulated gene 6.^4,11,24^ Some of these changes appeared to be related to longer term consequences of the injury, as elevated levels of MCP-1 and interleukin-6 at the time of the injury were independently associated with worse knee symptoms (assessed by the Knee injury and Osteoarthritis Outcome Score [KOOS]) including pain after 2 years.^25^

The balance between pro-inflammatory responses and the resolution of inflammation is influenced, at least in part, by the levels of resolution molecules derived from docosahexaenoic acid (DHA) and eicosapentaenoic acid (EPA) as well as the anti-inflammatory epoxyeicosatrienoic acids (EETs) produced from arachidonic acid (AA) (see Appendix Figure A1, available in the online version of this article). Associations have been reported between a key resolution pathway metabolite of DHA, 17–hydroxydocosahexaenoic acid (HDHA), with thermal pain thresholds in healthy volunteers and with pain scores in patients with knee osteoarthritis.^21^ In addition, metabolites of the soluble epoxide hydrolase (sEH) pathway (EETs and dihydroxyeicosatrienoic acids [DHETs]) are associated with osteoarthritis progression^22^ and measures of osteoarthritis pain,^7^ and polymorphisms in the sEH gene are also associated with osteoarthritis pain.^7^ The analgesic potential of targeting both the resolvin pathways^9^ and the sEH^7^ has been demonstrated in rodent models of posttraumatic osteoarthritis. Plasma levels of AA are associated with synovial inflammation in patients with osteoarthritis,^1^ and pro-resolving lipid mediators have also been detected in synovial fluid of patients with osteoarthritis.^10^ Overall, there is mounting evidence that the balance between pathways mediating inflammation and its resolution may contribute to the development of chronic osteoarthritis pain.

The aim of this study was to quantify the serum levels of a range of bioactive lipids longitudinally after an acute knee injury in otherwise healthy participants and compare these with the levels in age- and sex-matched controls. A subset of participants from the Knee Injury Cohort at the Kennedy (KICK) study, consisting of a longitudinal cohort of young, highly active participants who had experienced an acute knee injury, were used for this study. We hypothesized that systemic levels of bioactive lipids, which either drive or resolve inflammation, may be associated with knee symptoms at 3 time points during a 2-year period after recruitment to our study and that these levels would be different in those who had not experienced a knee injury.

## Methods

### Participants and Study Design

The KICK study used a prospective cohort design and recruited patients between 2010 and 2014 from a population exposed to an acute knee injury who underwent assessments at 6 hospitals and clinics in London, in the United Kingdom. Inclusion criteria were as follows: a clinically significant acute knee injury within 8 weeks of recruitment, age 16 to 50 years, knee effusion confirmed clinically or by magnetic resonance imaging, and evidence of ≥1 specified structural injuries on magnetic resonance imaging (meniscal tear, cruciate ligament rupture, collateral ligament tear, posterolateral corner injury, traumatic chondral defect, articular or periarticular fracture, patellofemoral dislocation, or tibiofemoral dislocation) within 8 weeks of the baseline visit. All participants gave written informed consent before screening according to the Declaration of Helsinki. Institutional review board approval was obtained (South East London Research Ethics Committee 5 [REC 10/H0706/44]). For the purposes of this study, KICK participants were included if they had serum samples and KOOS scores from 3 study visits: baseline, 3 months, and 2 years. There were 47 injured participants of a total of 150 who had both serum samples and KOOS scores at these time points. Baseline samples were collected at a median of 24 days (interquartile range, 3-57 days) after the injury, with following visits scheduled at 3 months and 2 years after the baseline visit. Although not a prerequisite or inclusion criterion, the majority of participants underwent surgery on their injured knee, which was performed after the baseline visit (typically immediately after), as part of their usual care.

Healthy participants gave consent for blood sampling (REC 11/H0711/17). They had no history of arthritis or knee pain and were matched, as far as possible, with injured participants by age and sex.

### Clinical Outcomes

The primary outcome measure in the KICK study was the KOOS, from which the KOOS-4, a single composite score, could be calculated (a mean of 4 of the 5 KOOS subscales including Pain, Symptoms, Sport/Recreation, and Quality of Life).^17^ The KOOS-4 is highly correlated with the presence of knee pain as well as associated symptoms.^5^ The KOOS and the Tegner score, a measure of activity that was an identified possible confounder,^3^ were each administered during the same 3 study visits as the samples were collected (Table 1; see also Appendix Figures A2 and A3, available online). A medical review, including medication history (including oral anti-inflammatory drugs and omega-3 supplementation), was undertaken at each visit. Other factors that were available and predefined as potentially influencing outcomes for this study included age at baseline, sex, body mass index (BMI), and Tegner score (Table 1).

**Table 1:** Baseline Characteristics of Study Participants^*a*^

	All Participants in KICK Study (N = 150)	Injured Participants in This Substudy (n = 47)	Healthy Controls (n = 33)
Age, y	25.0 (21.0-30.0)	28.0 (21.0-41.2)	33.7 (21.0-59.0)
BMI	25.0 (23.0-28.0)	24.8 (22.8-28.4)	—
Sex, male/female	123 (82.0)/27 (18.0)	34 (71.1)/13 (28.9)	18 (54.5)/15 (45.5)
Knee surgery	145 (96.7)	45 (95.7)	—
Time from injury to baseline, d	17 (1-57)	24 (3-57)	—
KOOS-4 score	44.4 (31.1-57.0)	39.7 (27.5-48.1)	—
Tegner score			
Before injury	10.0 (9.0-10.0)	8.5 (6.0-10.0)	—
Baseline	2.0 (1.0-3.0)	2.0 (1.0-3.0)	—
Injury type			
Meniscal tear	27 (18.0)	7 (14.9)	—
Single ligament rupture	28 (18.7)	8 (17.0)	—
ACL rupture + meniscal tear	61 (40.7)	26 (55.3)	—
Severe trauma^*b*^	34 (22.7)	6 (12.8)	—
Smoking status			
Current smoker	3 (2.0)	2 (4.3)	—
Former smoker (≤10 y)	2 (1.3)	0 (0.0)	—
Former smoker (>10 y)	8 (5.3)	4 (8.5)	—
Never smoked	133 (88.7)	41 (87.2)	—
Unknown	4 (2.7)	0 (0.0)	—
Type 2 diabetes	1 (0.7)	1 (2.1)	—

a Data are presented as median (IQR) or n (%). ACL, anterior cruciate ligament; BMI, body mass index; KICK, Knee Injury Cohort at the Kennedy; KOOS, Knee injury and Osteoarthritis Outcome Score. Dashes indicate data not available.

b Severe trauma was defined as a combined ligament (>1) rupture, fracture, or dislocation.

### Biosamples

Venous blood was collected from injured participants at each study visit and from healthy controls at a single visit in plain Vacutainer tubes (Becton Dickinson). Blood was allowed to clot for 40 minutes and then centrifuged at 1600*g* for 15 minutes at 20°C before aliquoting supernatants into cryovials and storing at −80°C in monitored freezers until analysis.

### Lipidomic Analysis

A total of 18 quality control (QC) thawed serum samples and calibration standards were spiked with internal standards and extracted via protein precipitation, followed by solid phase extraction. Extracts were reconstituted and chromatographically separated via ultra-high performance liquid chromatography on a reversed-phase C18 column. Analytes were detected using a triple quadrupole mass spectrometer (QTRAP 6500+; Sciex). The ratio of analyte peak area to internal standard peak area was used for quantification, which was deemed reliable if the coefficient of variation value across QC sample concentrations was <15%. This method was adapted from a previously reported method to include a larger number of analytes and updated instrumentation.^27^

### Statistical Analysis

At the outset of the project, we predefined the following research questions to be answered by our analyses:

1. Is an acute knee injury associated with changes in bioactive lipid mediators (compared with healthy age- and sex-matched control data)?
2. Do levels of bioactive lipid mediators change over time after an acute knee injury?
3. Are levels of bioactive lipid mediators associated with KOOS-4 scores at 3 time points over a 2-year period?
4. Are baseline levels of bioactive lipid mediators associated with future KOOS-4 scores after an acute knee injury?

The statistical analyses related to these 4 questions are outlined as follows:

1. Groups were assessed for a normal distribution. Differences between groups (compared with the healthy control group) were assessed using the Kruskal-Wallis test and corrected for multiple comparisons using the Dunn test.
2. Differences in levels between the time points in the same participant were evaluated using matched-pair analysis (Friedman test). Multivariate analysis using MetaboAnalyst (Version 4.0; https://www.metaboanalyst.ca/) was undertaken, and principal component analysis was performed for data visualization. Partial least squares discriminant analysis (PLS-DA) was performed to identify the key lipids involved in the difference between 2 predefined time points after the knee injury: baseline and 2 years. Cross-validation analysis was used to validate the developed PLS-DA model based on accuracy, *R*^2^ and *Q*^2^ values, and a permutation test. Lipids having variable importance in projection (VIP) scores >1 were considered to play a key role in cluster differentiation.
3 and 4. Associations between serum lipid levels and knee symptoms (KOOS-4) and potential covariates (age, sex, BMI, Tegner score, and days from injury to sampling [for baseline time point only]) were assessed by linear regression analyses using R software (www.r-project.org).

For all analyses, *P* values were corrected for multiple testing using the Bonferroni method. Graphs and descriptive analyses were produced using GraphPad Prism (Version 7; GraphPad Software).

Sensitivity analyses were performed, excluding those participants who were either supplementing with omega-3 or taking an oral nonsteroidal anti-inflammatory drug (NSAID) or cyclooxygenase-2 (COX-2) inhibitor (collectively referred to as oral anti-inflammatory drugs) at the time of blood sampling. As this was a hypothesis-generating study, no a priori sample size calculation was conducted (we included all eligible participants in the substudy following our criteria).

## Results

### Clinical Characteristics of Participants

**Figure 1.**
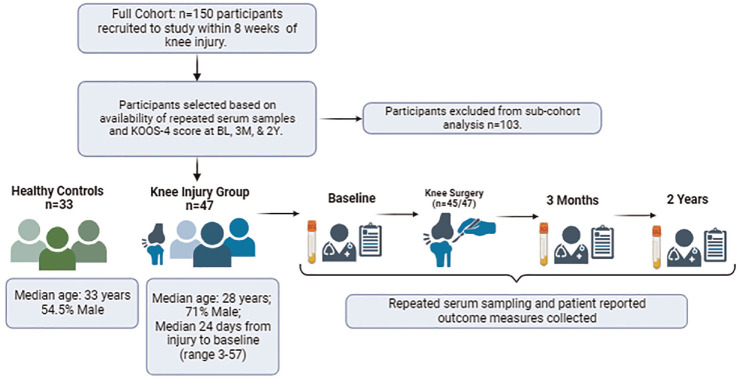
Graphical workflow of study design and data collection (bioredner.com). 2Y, 2 years; 3M, 3 months; BL, baseline; KOOS-4, Knee injury and Osteoarthritis Outcome Score–4.

Of 150 participants in the KICK cohort, recruited within a median of 24 days from their acute knee injury, 47 injured participants (median, 24 days from injury) met the criteria for this substudy and had their serial serum samples processed for lipidomic analysis (Figure 1).

The median age of this subcohort was 28 years, the majority were male, and participants were highly active before the injury, with similar characteristics to those of the overall cohort (Table 1). The majority of injured participants (45/47) underwent knee surgery during the study, which took place after baseline sample collection. The second visit was scheduled at 3 months after baseline. On review of available medical history, 1 participant had a diagnosis of type 2 diabetes, which was controlled with diet.

### Levels of Pro- and Anti-inflammatory Bioactive Lipids After Acute Knee Injury

**Figure 2. fig2-03635465241228209:**
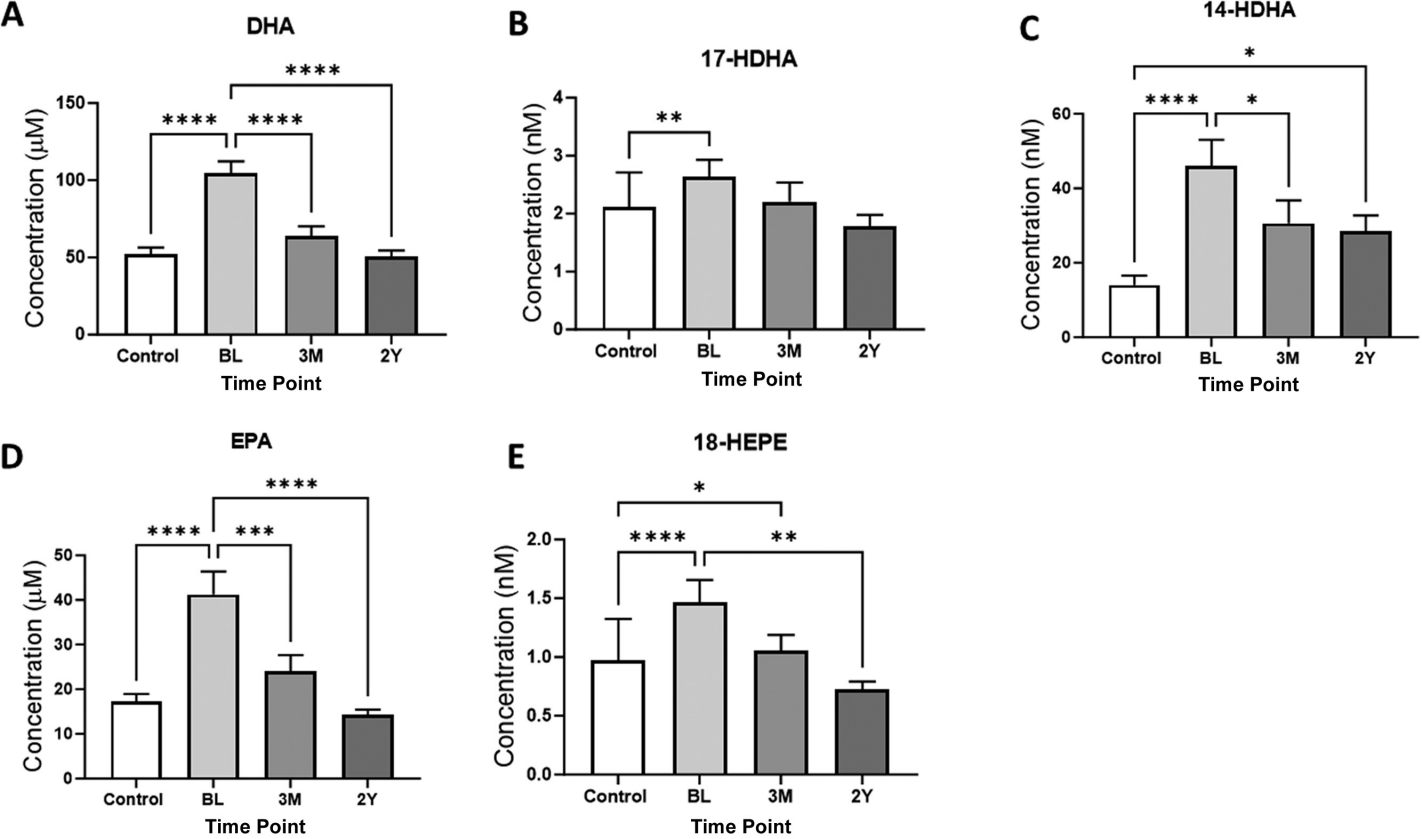
Levels of omega-3 polyunsaturated fatty acids: (A) docosahexaenoic acid (DHA), (B) 17–hydroxydocosahexaenoic acid (17-HDHA), (C) 14-HDHA, (D) eicosapentaenoic acid (EPA), and (E) 18–hydroxyeicosapentaenoic acid (18-HEPE) in controls (n = 33) and in injured participants (n = 47) at baseline (BL), 3 months, and 2 years. Graphs show mean ± SEM levels. Statistical analysis with the Kruskal-Wallis test, adjusted for multiple comparisons using the Dunn test. *P≤ .05, **P≤ .01, ***P≤ .001, ****P≤ .0001.

Levels of bioactive lipids were quantified in the injured participants’ sera from the 3 visits and in the healthy control samples (Table 2). A total of 41 lipid metabolites met bioanalytical QC criteria for further analysis. Levels of the omega-3 polyunsaturated fatty acids (PUFAs) DHA (*P*≤ .0001) and EPA (*P*≤ .0001), and their metabolites 14-HDHA and 18–hydroxyeicosapentaenoic acid (18-HEPE), were significantly higher in injured participants compared with healthy controls and at baseline compared with the later time points (Figure 2, A and C [Fig fig2-03635465241228209]-E). In addition, serum levels of the DHA metabolite 17-HDHA were also significantly higher at baseline in injured participants compared with healthy controls but at later time points were not significantly different from controls (Figure 2B). Any potential effect of concurrent omega-3 supplementation or oral anti-inflammatory medication use on these levels was considered. Sensitivity analyses were carried out excluding samples of participants who had supplemented and/or medicated within 1 month of the sample collection time point (for omega-3: baseline, n = 12; 3 months, n = 7; 2 years, n = 7) (for oral anti-inflammatory drugs: baseline, n = 28; 3 months, n = 13; 2 years, n = 8). Excluding these participants did not change our findings (data not shown).

**Table 2:** Quantification of Bioactive Lipids in Study Participants’ Sera^*a*^

	Healthy Controls (n = 33)	Injured Participants		
		Baseline (n = 47)	3 mo (n = 47)	2 y (n = 47)
5,6-EET, nM	0.53 ± 0.26	0.42 ± 0.14	0.33 ± 0.11	0.31 ± 0.11
8,9-EET, nM	2.02 ± 5.33	0.95 ± 0.57	0.90 ± 0.85	0.84 ± 0.58
11,12-EET, nM	133 ± 150	265 ± 224	247 ± 231	269 ± 257
14,15-EET, nM	0.63 ± 0.31	0.64 ± 0.24	0.43 ± 0.20	0.39 ± 0.19
5,6-DHET, nM	0.72 ± 0.82	0.53 ± 0.31	0.36 ± 0.23	0.33 ± 0.16
8,9-DHET, nM	0.26 ± 0.13	0.32 ± 0.24	0.24 ± 0.12	0.22 ± 0.09
11,12-DHET, nM	0.58 ± 0.17	0.94 ± 0.52	0.60 ± 0.30	0.58 ± 0.21
14,15-DHET, nM	0.51 ± 0.13	0.76 ± 0.42	0.54 ± 0.25	0.52 ± 0.15
5,6-ratio	1.13 ± 0.74	0.93 ± 0.45	1.12 ± 0.51	1.05 ± 0.40
8,9-ratio	5.76 ± 10.93	3.69 ± 2.70	4.24 ± 4.08	4.00 ± 2.24
11,12-ratio	227 ± 247	353 ± 318	472 ± 526	495 ± 479
14,15-ratio	1.24 ± 0.53	0.95 ± 0.36	0.83 ± 0.35	0.77 ± 0.30
TXB2, nM	39.7 ± 43.1	28.7 ± 23.4	57.4 ± 52.1	53.0 ± 46.4
11-dehydro-TXB2, nM	0.48 ± 0.64	0.57 ± 0.67	1.20 ± 1.24	1.25 ± 1.28
PGE2, nM	0.44 ± 0.50	0.59 ± 0.46	0.85 ± 0.73	0.83 ± 0.72
PGD2, nM	0.43 ± 0.72	0.11 ± 0.06	0.20 ± 0.13	0.18 ± 0.12
LTB4, nM	1.56 ± 2.25	0.65 ± 0.94	2.03 ± 3.00	1.93 ± 2.72
16-HETE, nM	0.24 ± 0.06	0.25 ± 0.07	0.23 ± 0.08	0.23 ± 0.06
11-HETE, nM	4.36 ± 7.79	2.05 ± 1.46	3.14 ± 2.56	2.88 ± 2.24
15-HETE, nM	12.2 ± 19.5	8.5 ± 6.0	10.6 ± 8.2	10.8 ± 9.1
8-HETE, nM	2.05 ± 5.34	1.01 ± 0.62	0.98 ± 0.92	0.93 ± 0.68
12-HETE, nM	130 ± 142	270 ± 220	245 ± 220	269 ± 248
5-HETE, nM	20.10 ± 71.00	1.57 ± 0.85	2.78 ± 7.09	1.66 ± 1.49
12-HpETE, nM	3.11 ± 1.70	2.72 ± 1.24	3.09 ± 2.23	3.33 ± 2.20
13-OxoODE, nM	22.70 ± 10.50	31.20 ± 21.00	18.00 ± 8.30	16.60 ± 7.63
13-HODE, nM	59.40 ± 45.80	61.40 ± 42.20	64.14 ± 116.90	42.60 ± 24.90
9-OxoODE, nM	2.68 ± 1.53	3.87 ± 4.81	2.24 ± 0.95	1.81 ± 0.63
9-HODE, nM	25.50 ± 27.60	22.20 ± 17.70	19.60 ± 16.90	16.10 ± 9.65
18-HEPE, nM	0.97 ± 2.00	1.46 ± 1.30	1.05 ± 0.91	0.72 ± 0.48
17-HDHA, nM	2.12 ± 3.33	2.63 ± 2.02	2.21 ± 2.25	1.78 ± 1.32
14-HDHA, nM	14.0 ± 14.5	46.0 ± 48.1	30.6 ± 41.7	28.6 ± 28.1
5,12-DiHETE, nM	1.59 ± 2.33	0.75 ± 1.06	2.23 ± 3.42	2.21 ± 3.23
8,9-DiHETE, nM	0.06 ± 0.04	0.12 ± 0.08	0.10 ± 0.12	0.06 ± 0.04
2-AG, nM	57.3 ± 46.5	30.7 ± 28.4	38.0 ± 24.7	31.4 ± 24.8
Anandamide, nM	0.61 ± 0.36	0.62 ± 0.26	0.44 ± 0.21	0.49 ± 0.21
Oleoylethanolamide, nM	2.97 ± 0.93	3.37 ± 1.16	2.74 ± 0.93	2.93 ± 0.72
Palmitoylethanolamide, nM	7.85 ± 1.45	9.85 ± 2.96	8.36 ± 2.34	8.57 ± 2.23
Linoleic acid, µM	273 ± 113	690 ± 289	344 ± 229	278 ± 122
AA, µM	70.6 ± 29.7	108.0 ± 33.4	74.5 ± 27.9	68.0 ± 22.5
EPA, µM	17.30 ± 9.32	41.20 ± 35.00	24.20 ± 24.00	14.20 ± 8.23
DHA, µM	52.3 ± 23.5	104.6 ± 51.8	64.0 ± 42.1	50.3 ± 27.8

a Data are presented as mean ± SD. Measurements for a panel of 41 lipids meeting prespecified quality control requirements are shown, quantified by liquid chromatography–tandem mass spectrometry. 2-AG, 2-arachidonoylglycerol; AA, arachidonic acid; DHA, docosahexaenoic acid; DHET, dihydroxyeicosatrienoic acid; DiHETE, dihydroxyeicosatetraenoic acid; EET, epoxyeicosatrienoic acid; EPA, eicosapentaenoic acid; HDHA, hydroxydocosahexaenoic acid; HEPE, hydroxyeicosapentaenoic acid; HETE, hydroxyeicosatetraenoic acid; HODE, hydroxyoctadecadienoic acid; HpETE, hydroperoxyeicosatetraenoic acid; LTB4, leukotriene B4; OxoODE, oxo-octadecadienoic acid; PGD2, prostaglandin D2; PGE2, prostaglandin E2; TXB2, thromboxane B2.

**Figure 3.**
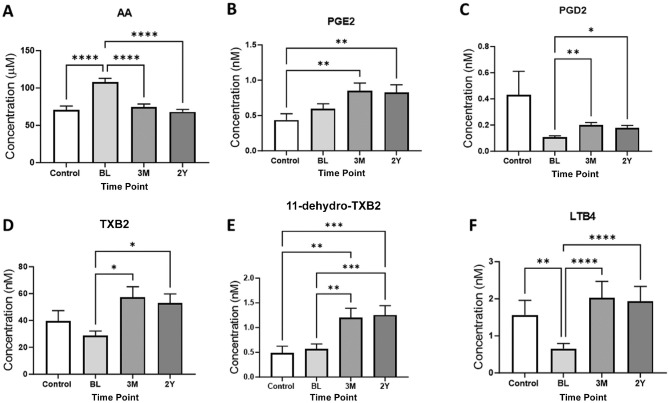
Levels of omega-6 polyunsaturated fatty acids: (A) arachidonic acid (AA) and its metabolites, (B) prostaglandin E2 (PGE2), (C) prostaglandin D2 (PGD2), (D) thromboxane B2 (TXB2), (E) 11–dehydrothromboxane B2 (11-dehydro-TXB2), and (F) leukotriene B4 (LTB4) in controls (n = 33) and in injured participants (n = 47) at baseline (BL), 3 months (3M), and 2 years (2Y). Graphs show mean ± SEM levels. Statistical analysis with the Kruskal-Wallis test, adjusted for multiple comparisons using the Dunn test. *P < .05, **P < .01, ***P < .001, ****P < .0001.

Serum levels of AA were also higher in the injured participants at baseline compared with healthy controls and compared with later time points (Figure 3A). Despite this, there were no differences in the serum levels of the AA-derived lipids prostaglandin E2 (PGE2), prostaglandin D2 (PGD2), thromboxane B2 (TXB2), and 11-dehydrothromboxane B2 (11-dehydro-TXB2) in injured participants at baseline compared with the healthy controls (Figure 3, B-E). However, at 3 months and 2 years, serum levels of PGE2 were significantly higher in injured participants than in healthy controls (Figure 3B). In addition, after the knee injury, levels of PGD2 and TXB2 were significantly higher at 3 months and 2 years compared with baseline levels (Figure 3, C and D). Other differences of note were significantly higher serum levels of 11-dehydro-TXB2 at 3 months and 2 years in injured participants compared with baseline levels and healthy controls (Figure 3E). Finally, levels of leukotriene B4 (LTB4) were significantly lower in injured participants at baseline compared with the later time points and compared with levels in healthy controls (Figure 3F).

**Figure 4.**
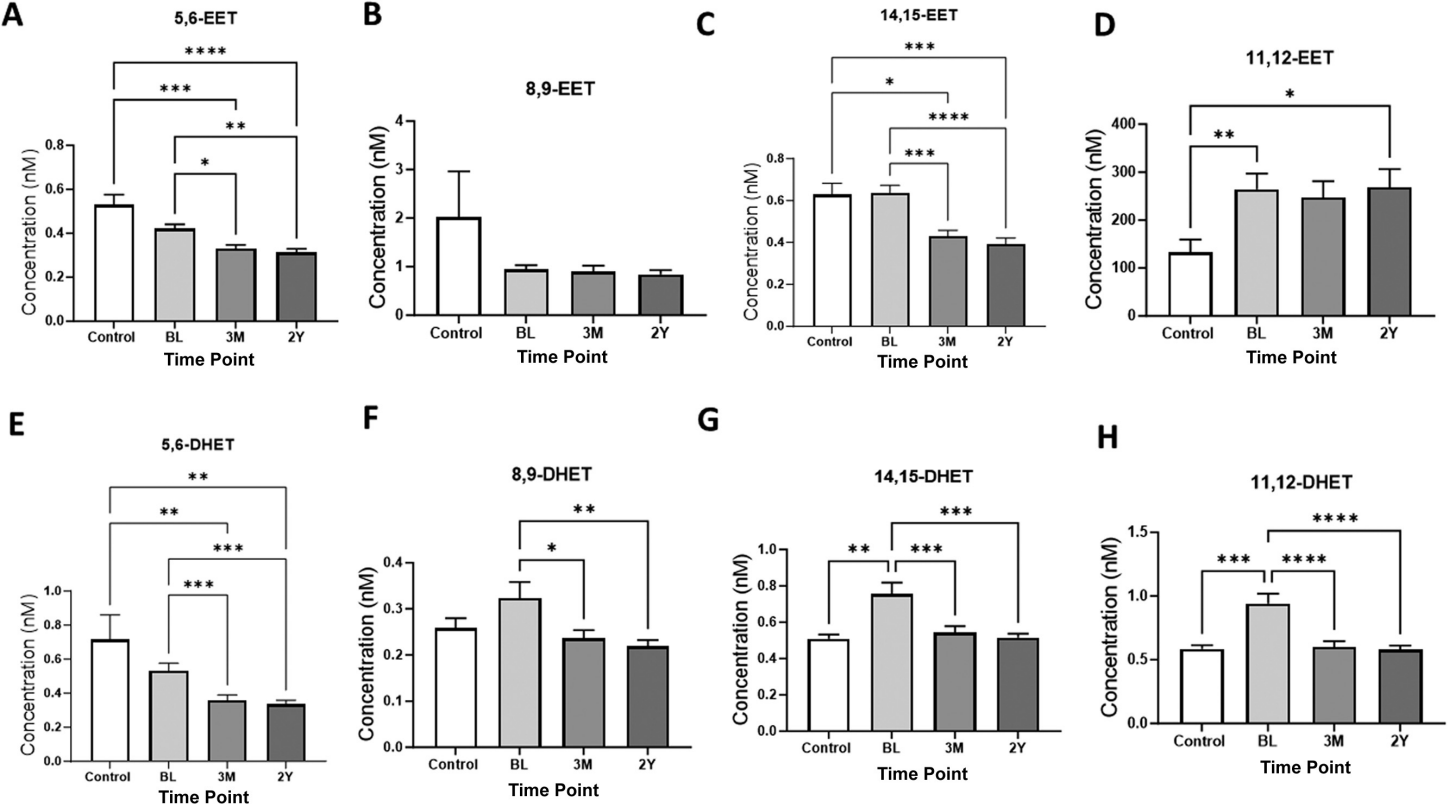
Levels of arachidonic acid metabolites—(A) 5,6–epoxyeicosatrienoic acid (EET), (B) 8,9-EET, (C) 14,15-EET, and (D) 11,12-EET—and corresponding soluble epoxide hydrolase metabolites, (E) 5,6–dihydroxyeicosatrienoic acid (DHET), (F) 8,9-DHET, (G) 14,15-DHET, and (H) 11,12-DHET—in controls (n = 33) and in injured participants (n = 47) at baseline (BL), 3 months (3M), and 2 years (2Y). Graphs show mean ± SEM levels. Statistical analysis with the Kruskal-Wallis test, adjusted for multiple comparisons using the Dunn test. *P≤ .05, **P≤ .01, ***P≤ .001, ****P≤ .0001.

There were also significant differences in levels of the anti-inflammatory lipids EETs and their metabolites DHETs. At all time points, levels of 5,6-EET were lower in those with injuries compared with healthy controls (Figure 4A), with a similar pattern evident for 8,9-EET and 14,15-EET at the 3-month and 2-year time points (Figure 4, B and C). By contrast, levels of 11,12-EET were higher at all time points in patients with knee injuries compared with the healthy controls (Figure 4D). Levels of 5,6-DHET, which is downstream of 5,6-EET, were lower in injured participants than in healthy controls at all time points (Figure 4E). There were also significantly higher levels of 8,9-DHET at baseline in injured participants compared with samples at 3 months and 2 years as well as compared with the healthy controls (Figure 4F). Levels of 11,12- and 14,15-DHET were also significantly higher at baseline in injured participants compared with healthy controls (Figure 4, G and H).

There were some minor differences in levels of the endocannabinoid anandamide and related lipids (oleoylethanolamide and palmitoylethanolamide) between the groups but no clear overall trend (Table 2). By contrast, levels of 2-arachidonoylglycerol were significantly lower at all time points in the injured group compared with healthy controls (Table 2).

The levels of the linoleic acid metabolites 9– and 13–hydroxyoctadecadienoic acid were stable across both groups, with the exception of lower levels of 13–hydroxyoctadecadienoic acid in the injured group at 2 years compared with levels at baseline (Table 2). Their respective downstream metabolites 9– and 13–oxo-octadecadienoic acid (OxoODE) showed higher levels at baseline in the injured group compared with the later time points and compared with the control group (Table 2).

### Partial Least Squares Discriminant Analysis

**Figure 5.**
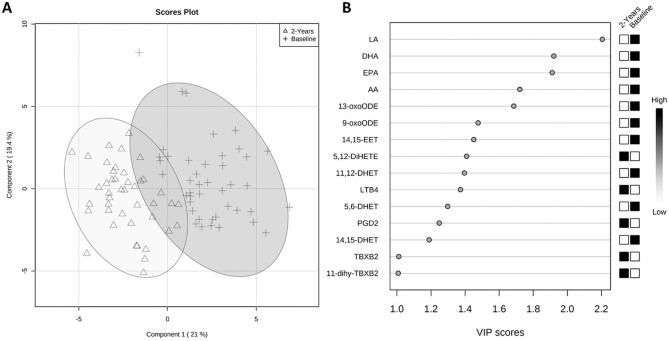
(A) Partial least squares discriminant analysis of serum lipids in injured participants (n = 47) showing 2 different clusters at 2 different time points (baseline and 2 years), with *R*^2^ = 0.77, *Q*^2^ = 0.70, and accuracy = 0.95. (B) The 15 lipids with variable importance in projection (VIP) scores >1.0, considered as being involved in the difference between the 2 time points.

PLS-DA revealed a clear difference in the serum lipid profiles between samples taken at baseline and 2 years, showing 2 different clusters (Figure 5A). The key lipids contributing to the difference were ranked by the VIP score (Figure 5B). Overall, 15 different lipids had a VIP score >1.0 and were identified as the most important lipid mediators involved in the difference between the 2 groups, with the PUFAs ranked highest. Apart from the PUFAs, the other top ranked contributing lipids were mainly pro-inflammatory mediators, such as OxoODEs (9- and 13-OxoODE), DHETs (5,6-, 11,12-, and 14,15-DHET), thromboxanes (TXB2 and 11-dehydro-TXB2), PGD2, LTB4, and 5,12–dihydroxyeicosatetraenoic acid. The outcomes of this multivariate analysis were consistent with univariate analyses of the lipid levels between the 2 groups.

### Levels of DHETs and Associations With Persistent Knee Symptoms at 2 Years

**Figure 6.**
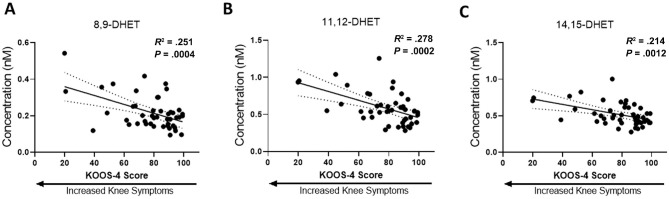
Associations between Knee injury and Osteoarthritis Outcome Score (KOOS)–4 scores and levels of (A) 8,9–dihydroxyeicosatrienoic acid (DHET), (B) 11,12-DHET, and (C) 14,15-DHET at 2 years in injured participants (n = 47) by linear regression analyses. Dotted lines represent 95% CIs.

Univariate linear regression analyses were carried out to assess potential associations between serum bioactive lipid levels and knee symptoms evaluated by the KOOS-4 at each time point. At 2 years, there was an association between higher levels of 8,9-, 11,12-, and 14,15-DHET and worse knee symptoms (lower KOOS-4 score), which remained after adjusting for multiple testing (Figure 6). Although these associations may have been driven by unadjusted confounders such as age, sex, BMI, Tegner score, and days from injury, exploratory univariate analyses to test for any associations between lipid levels and these clinical factors did not support a contribution. Significant associations were observed between 2-arachidonoylglycerol and BMI at all time points as well as DHA, 5,6-DHET, and 8,9-DHET with BMI at the 3-month time point only. There were no associations between baseline lipid levels and days between the injury and baseline sampling (see Appendix Table A1, available online).

When considering the clinical trajectory of a knee injury in this subset of injured participants, it was clear that, as for the cohort as a whole, there was a wide range of changes in symptoms over the 2 years, with the majority of participants showing clinical recovery, demonstrated by a general improvement in KOOS-4 scores and increased levels of activity (Table 2; see also Appendix Figures A2 and A3, available online). We investigated whether any of the lipids measured at baseline may predict future knee pain at 2 years. In this substudy, there were no significant associations between serum lipid levels at baseline and KOOS-4 scores at 2 years.

## Discussion

The major finding of this study in young adults was that after an acute knee injury, there was a marked increase in the levels of substrates for the 2 main resolution pathways, DHA and EPA, in the majority of participants. These levels then declined at 3 months and 2 years after the injury. Changes were not restricted to the DHA/EPA pathways, as levels of some EETs and their corresponding DHET metabolites, which comprise another major anti-inflammatory pathway, were also altered at baseline in the majority of injured participants. EETs are derived from AA, and at baseline, levels of AA were also elevated in the injured group. Interestingly, it was only after months to years following the injury that levels of AA-derived pro-inflammatory molecules such as PGE2 and PGD2 were elevated in the majority of participants. Levels of bioactive lipids quantified at baseline were not associated with subsequent knee symptoms at 2 years of follow-up in this cohort. However, higher levels of 8,9-, 11,12-, and 14,15-DHET were cross-sectionally associated with having worse knee symptoms at 2 years.

Our observation of increased levels of the omega-3 PUFAs DHA and EPA and their metabolites (which are the precursors for the D- and E-series resolution molecules^18^) within 8 weeks of an acute knee injury suggests that the biological response to this type of injury may include the activation of these pro-resolution pathways. Such pathways have been reported in other contexts to counter pro-inflammatory signaling and restore tissue homeostasis.^2^ The profile of changes in levels of the anti-inflammatory EETs in patients after a joint injury at baseline compared with healthy controls and at later time points was more variable than that of the DHA/EPA pathway. It was notable that levels of DHETs (metabolites of the EETs generated by sEH) were more consistently altered between the different members of the EET family, with significant increases in levels of 8,9-, 11,12-, and 14,15-DHET at baseline in injured participants compared with later time points and in some cases also when compared with levels in healthy controls. At 2 years, levels of 8,9-, 11,12-, and 14,15-DHET were significantly associated with worse knee symptoms. Previously, we reported an association between knee osteoarthritis progression and levels of 8,9-, 11,12-, and 14,15-DHET^22^ as well as positive correlations/associations with osteoarthritis knee pain.^7^ Although we cannot infer any causation, our data lend further support to a potential role of the sEH pathway in the persistence of knee pain. Experimental studies also support these clinical observations and have shown that the inhibition of sEH reduces pain behavior in a slow-progressing mouse model of posttraumatic osteoarthritis (surgical destabilization of the medial meniscus) and decreases circulating levels of the DHETs.^7^ Importantly, this target has also been confirmed as having therapeutic potential in canine osteoarthritis in which inhibiting the sEH pathway significantly reduced pain behavior in treated animals^15^ as well as in a rodent albumin-induced arthritis model.^20^ The findings of the present study support further investigation of these pathways after an acute knee injury and suggest that the outcome of current clinical trials of an sEH inhibitor for neuropathic pain^8^ may have wider relevance.

Levels of the classic pro-inflammatory metabolites of AA, including PGE2, PGD2, TXB2, 11-dehydro-TXB2, and LTB4, were reduced or unchanged at baseline in injured participants compared with healthy controls, and then levels were elevated at later time points after the knee injury. Collectively, our data provide systemic evidence for the activation of pro-resolution pathways and deactivation of the pro-inflammatory pathways at early time points after an injury, which if mirrored at the site of the injury may aid tissue recovery. Previous studies have identified significantly lower serum levels of pro-inflammatory molecules (MCP-1, activin A, matrix metalloproteinase 3, tissue inhibitor of metalloproteinases 1, and tumor necrosis factor–stimulated gene 6) in patients at baseline compared with healthy controls.^25^ At the level of the joint, however, synovial fluid levels of these pro-inflammatory molecules were significantly raised at baseline. Improved understanding of the relationship between synovial fluid, tissue, and systemic levels of these molecules is needed, including whether these levels are independent of one another or if a temporal or reciprocal relationship exists between circulating and tissue environments in the overall inflammatory/resolution response. Follow-up studies examining the relationships of these molecules with paired synovial fluid would be warranted to understand this process.

The findings of our univariate analyses were supported by PLS-DA of the entire data set, which indicated that the main molecules driving the difference in lipid profiles between samples collected at baseline versus 2 years from the same participants were the omega-3 and -6 PUFAs and a number of pro- and anti-inflammatory metabolites of AA and linoleic acid including several metabolites derived via the sEH pathway. The trajectory of recovery in the injured participants may at least in part be mediated by the magnitude of the activation of the resolution pathways, although this would require confirmation in a larger independent study.

The limitations of this study include a modest sample size (albeit with longitudinal resampling of the same participants) and some sex imbalance between the injury and control groups. Nevertheless, there were no substantial sex differences in the levels of these bioactive lipids based on our work. Although BMI data were collected and investigated as a potential confounder, the participants in this study were highly active, with many having a high muscle mass, which means that a high BMI is unlikely to have been caused by obesity. A subset of participants who attended all 3 study visits for blood sampling were selected for this study and therefore could have introduced some biases such as attrition bias and differential follow-up bias. For example, it is possible that participants attending all visits were more likely to have knee symptoms. However, we showed that our subcohort still exhibited diversity in the range of clinical outcomes at 2 years and that the baseline characteristics of this subcohort were largely similar to those of the overall cohort.^5^ Although longitudinal matched-pair analysis strengthened our statistical analysis, we cannot rule out the potential for confounding, such as differences between the injured and control groups in dietary factors, as this information was not collected. It is also of note that the control group could not be matched on physical activity and, given the nature of the injured participants, the two groups were likely to differ substantially in terms of physical activity levels. In addition, although the final time point of 2 years allowed the identification of participants with a substantial recovery in activity levels and improved knee symptoms as well as those who had not recovered clinically, assessments at later time points are required to understand the relationship between these pathways and the development of osteoarthritis after an injury. Because of the limited sample size, we did not adjust analyses for multiple confounders but performed predefined and post hoc sensitivity analyses. These did not identify any change from the reported results. We did not seek to examine structural changes. Because we did not know what we would find, we did not predefine more complex time-dependent repeated-measures analyses in what was an exploratory post hoc study in this cohort. The pathways that we have studied are affected, at least in part, by COX-2 activity, and therefore, the use of oral anti-inflammatory drugs is an important consideration. However, the sensitivity analysis excluding participants who were using NSAIDs did not alter the findings of our study. Nevertheless, there were a high number of participants using NSAIDs at baseline, which could have contributed to the complexity of changes between the individual EET/DHET molecules (as some EETs are metabolized by both sEH and COX-2).^16^ It is also of note that all except 2 participants underwent knee surgery after the baseline visit, which is a feature of this cohort (and many joint injury cohorts) and needs to be considered when interpreting 3-month and 2-year levels as a response to both the knee injury and the related surgical intervention. A surgical treatment pathway is at least relatively homogeneous in terms of this exposure within this cohort, but may mean that our findings are not generalizable to the population that sustains a knee injury but does not undergo surgery.

In conclusion, the profile of pro-resolving versus pro-inflammatory lipids at baseline suggests an initial activation of pro-resolution pathways, followed by the later activation of pro-inflammatory pathways. The association of systemic levels of EET/DHET with increased knee symptoms at 2 years adds to the mounting clinical evidence for a role of the sEH pathway in knee pain and supports the possibility of a therapeutic potential for targeting this pathway to reduce future knee pain.

## Supplemental Material

sj-pdf-1-ajs-10.1177_03635465241228209 – Supplemental material for The Effect of Acute Knee Injuries and Related Knee Surgery on Serum Levels of Pro- and Anti-inflammatory Lipid Mediators and Their Associations With Knee Symptoms Supplemental material, sj-pdf-1-ajs-10.1177_03635465241228209 for The Effect of Acute Knee Injuries and Related Knee Surgery on Serum Levels of Pro- and Anti-inflammatory Lipid Mediators and Their Associations With Knee Symptoms by James Turnbull, Rakesh R. Jha, David A. Barrett, Ana M. Valdes, Jennifer Alderson, Andrew Williams, Tonia L. Vincent, Fiona E. Watt and Victoria Chapman in The American Journal of Sports Medicine
