# Supplementary material for: The Effect of Acute Knee Injuries and Related Knee Surgery on Serum Levels of Pro- and Anti-inflammatory Lipid Mediators and Their Associations With Knee Symptoms
Source: Am J Sports Med. 2024 Feb 26;52(4):987–97. doi: 10.1177/03635465241228209 (PMC10943603; doi:10.1177/03635465241228209)
Supplement: sj-pdf-1-ajs-10.1177_03635465241228209 – Supplemental material for The Effect of Acute Knee Injuries and Related Knee Surgery on Serum Levels of Pro- and Anti-inflammatory Lipid Mediators and Their Associations With Knee Symptoms [file sj-pdf-1-ajs-10.1177_03635465241228209.pdf]

The effect of acute knee injury and related knee surgery in young active adults on serum levels of pro- and anti-inflammatory lipid mediators and their associations with knee symptoms.

Appendix

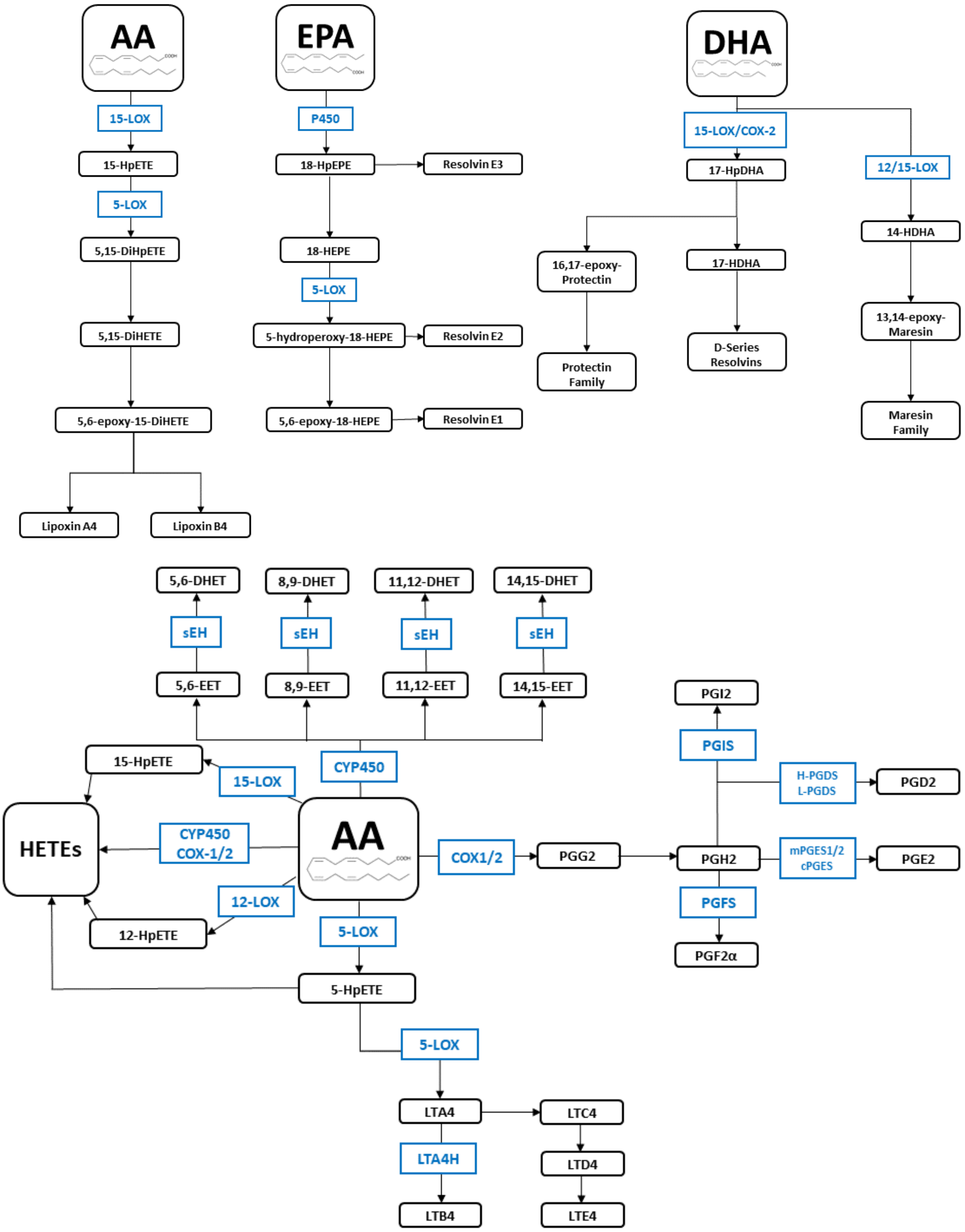

Figure A1. Overview of key lipid pathways and enzymes involved in metabolism of omega-3 and omega-6 polyunsaturated fatty acids. AA, arachidonic acid; AEA, anandamide; DHA, docosahexaenoic acid; DHET, dihydroxyeicosatrienoic acid; EET, epoxyeicosatrienoic acid; EPA, eicosapentaenoic acid; HDHA, hydroxydocosahexaenoic acid; HEPE, hydroxyeicosapentaenoic acid;

HETE, hydroxyeicosatetraenoic acid; HODE, hydroxyoctadecadienoic acid; LA, linoleic acid; LTB4, Leukotriene B4; OEA, oleoyl ethanolamide; oxoODE, oxo-octadecadienoic acid; PEA, palmitoyl ethanolamide; PGD2, prostaglandin D2; PGE2, prostaglandin E2; SD, standard deviation; TXB2, Thromboxane B2.

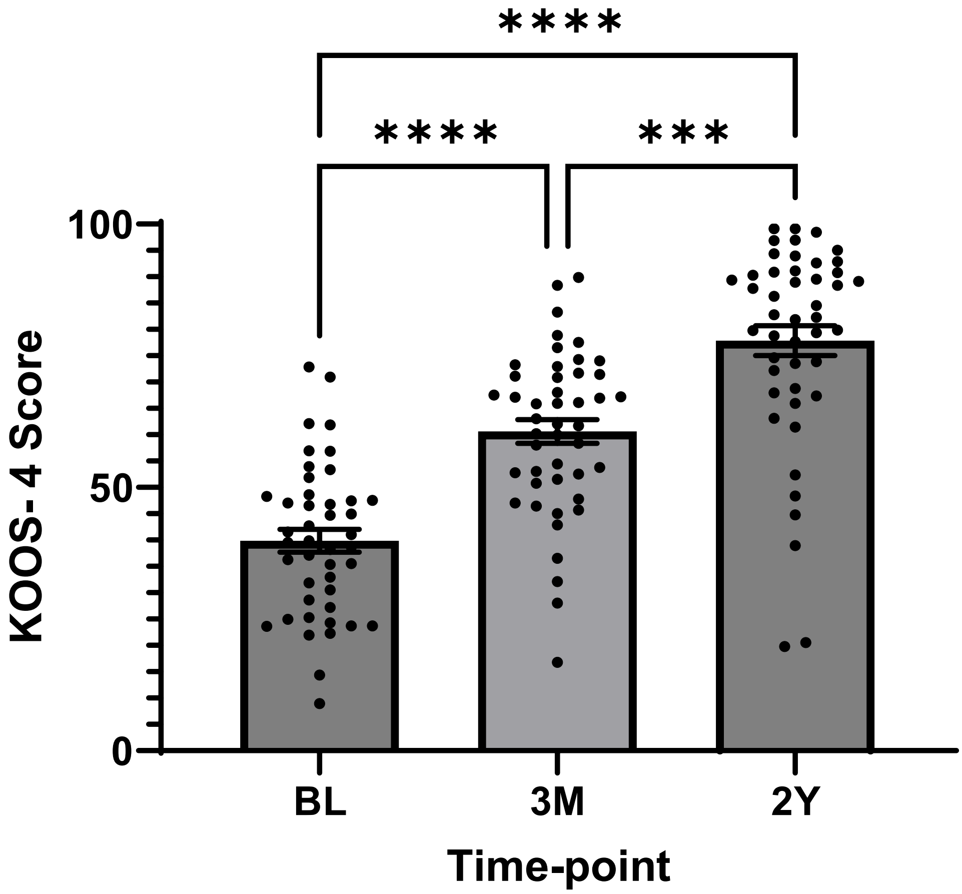

*Figure A2. Knee injury and osteoarthritis outcomes scores (KOOS-4) collected at baseline; 3 months; and 2 years in the 47 participants from the KICK study used in this sub-study. Lower KOOS-4 score denotes more severe knee symptoms. Data are presented as individual values with mean ± SEM error bars. Significance between time-points was assessed using a Kruskal - Wallis test, adjusted using Dunn's for multiple comparisons. *** <0.001, **** <0.0001.*

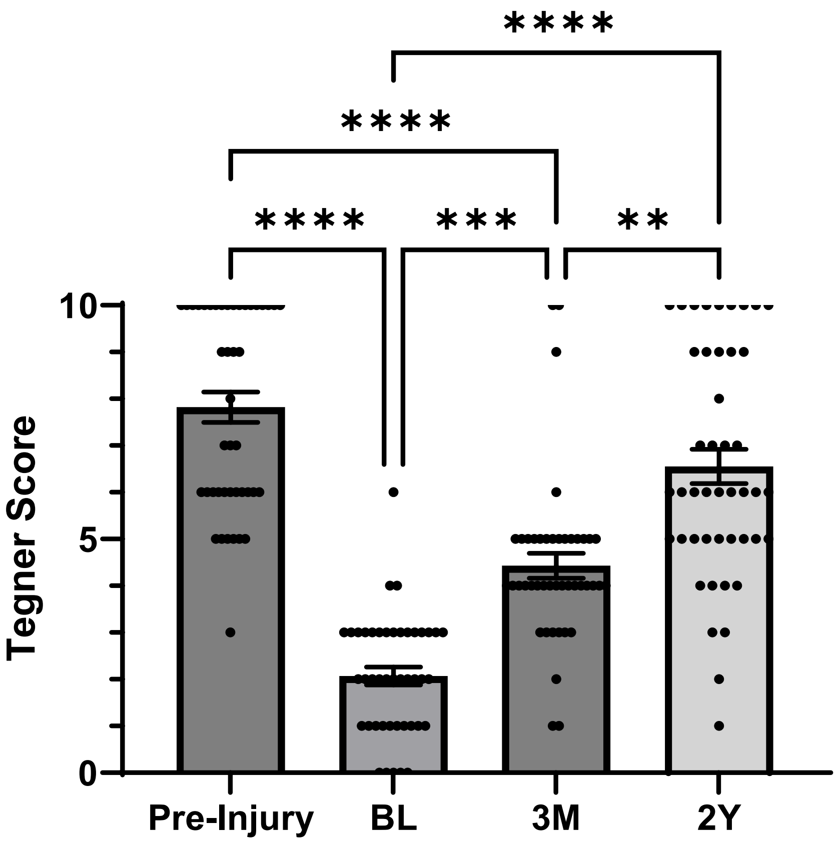

*Figure A3. Tegner scores collected at baseline; 3 months; and 2 years in the KICK sub-cohort used in this study. Higher Tegner score denotes higher levels of activity. Data are presented as individual values with mean ± SEM error bars. Significance between time-points were assessed using a Kruskal - Wallis test, adjusted using Dunn's for multiple comparisons. ** p=<0.01, *** p=<0.001, **** p=<0.0001.*

Table A1. Associations between serum lipid levels immediately post-injury with age, sex, Tegner score, and days from injury tested by univariate linear regression analysis between lipid levels and Tegner scores at 3 months and 2 years post-injury. Adjusting for multiple tests, statistical significance was assessed as p=<0.0012.

Lipid	Age		Sex		BMI (Baseline)		BMI (3 Months)		BMI (2 Years)		Tegner (Baseline)		Tegner (3 Months)		Tegner (2 Years)		Days from Injury (Baseline)	
	R squared	P value	R squared	P value	R squared	P value	R squared	P value	R squared	P value	R squared	P value	R squared	P value	R squared	P value	R squared	P value
**5,6-EET**	0.004	0.6830	0.001	0.8148	0.095	0.0395	0.117	0.0229	0.155	0.0082	0.011	0.4945	7.0E-05	0.9570	0.119	0.0205	0.003	0.6975
**8,9-EET**	0.002	0.7758	0.045	0.1667	0.004	0.6718	0.040	0.1938	0.001	0.8338	0.021	0.3511	0.002	0.7637	0.011	0.4905	0.008	0.5488
**11,12-EET**	1.89E-07	0.9978	0.043	0.1761	0.002	0.7531	0.002	0.7637	0.006	0.6070	0.009	0.5342	0.002	0.7961	0.010	0.5080	0.000	0.9296
**14,15-EET**	0.016	0.4188	0.012	0.4823	0.044	0.1595	0.156	0.0079	0.021	0.3535	0.049	0.1488	4.28E-04	0.8940	0.091	0.0445	0.001	0.8751
**5,6-DHET**	0.121	0.0209	0.036	0.2150	0.018	0.3680	0.291	0.0002	0.076	0.0703	0.008	0.5704	9.92E-04	0.8392	0.036	0.2124	0.014	0.4306
**8,9-DHET**	0.053	0.1320	0.025	0.3028	0.003	0.7067	0.222	0.0012	0.016	0.4063	0.052	0.1358	0.001	0.8033	0.015	0.4252	0.002	0.7553
**11,12-DHET**	0.083	0.0580	0.020	0.3624	0.009	0.5314	0.138	0.0129	0.013	0.4684	0.108	0.0295	0.012	0.4710	0.022	0.3271	0.006	0.5932
**14,15-DHET**	0.076	0.0702	0.019	0.3697	0.006	0.6200	0.120	0.0215	0.010	0.5088	0.091	0.0466	0.014	0.4443	8.85E-04	0.8462	0.004	0.6783
**5,6-Ratio**	0.131	0.0159	0.007	0.5956	0.024	0.3083	0.093	0.0444	0.001	0.8207	0.017	0.3988	0.004	0.6766	0.018	0.3811	0.059	0.0997
**8,9-Ratio**	0.023	0.3270	0.002	0.7748	0.014	0.4369	0.003	0.7111	3.20E-04	0.9082	0.036	0.2146	0.007	0.5990	0.016	0.4056	0.007	0.588
**11,12-Ratio**	0.021	0.3469	0.027	0.2893	0.012	0.4711	0.002	0.7660	0.008	0.5597	0.016	0.4080	0.005	0.6550	0.008	0.5475	0.000	0.9857
**14,15-Ratio**	0.066	0.0931	7.96E-04	0.8558	0.042	0.1741	0.034	0.2292	0.013	0.4698	0.016	0.4140	6.22E-04	0.8723	0.151	0.0084	0.001	0.8276
**TBXB2**	0.029	0.2665	0.004	0.6926	0.033	0.2257	6.33E-05	0.9591	0.019	0.3717	0.010	0.5224	0.009	0.5326	0.007	0.5949	0.005	0.643
**11-dehy-TBXB2**	1.36E-04	0.9401	0.028	0.2794	0.013	0.4631	0.009	0.5732	0.011	0.5009	0.036	0.2193	0.004	0.6939	6.61E-04	0.8669	0.002	0.7913
**PGE2**	0.008	0.5622	0.010	0.5190	8.04E-04	0.8621	0.005	0.6632	0.005	0.6702	0.008	0.5594	0.020	0.3544	0.009	0.5404	0.006	0.6356
**PGD2**	0.037	0.2090	0.109	0.0286	0.004	0.7437	0.009	0.5595	0.017	0.4184	0.009	0.5374	0.010	0.5177	0.005	0.6364	0.033	0.3403
**LTB4**	0.059	0.1132	0.100	0.0367	0.022	0.3317	4.41E-04	0.8924	0.020	0.3616	0.049	0.1475	0.018	0.3825	0.010	0.5138	0.000	0.9788
**16-HETE**	0.121	0.0207	0.013	0.4542	4.17E-04	0.8928	0.011	0.4933	1.61E-04	0.9348	0.171	0.0053	2.38E-07	0.9975	0.010	0.5165	0.011	0.4757
**11-HETE**	0.025	0.3089	0.009	0.5511	0.027	0.2751	0.005	0.6498	0.009	0.5503	0.017	0.4055	0.017	0.4036	5.44E-04	0.8792	0.002	0.7583
**15-HETE**	0.003	0.7105	0.025	0.3040	0.016	0.3981	0.007	0.5935	0.008	0.5576	0.016	0.4195	0.013	0.4664	0.004	0.6666	0.002	0.7503
**8-HETE**	3.06E-04	0.9102	0.058	0.1155	0.004	0.6764	0.036	0.2182	0.001	0.8294	0.020	0.3609	0.002	0.7549	0.009	0.5381	0.010	0.5052
**12-HETE**	3.63E-05	0.9690	0.048	0.1516	0.001	0.8172	0.002	0.7732	0.005	0.6482	0.011	0.4928	0.002	0.7712	0.012	0.4713	0.001	0.8528
**5-HETE**	6.55E-04	0.8691	0.028	0.2795	0.008	0.5447	0.046	0.1628	0.036	0.2178	0.035	0.2267	0.005	0.6630	0.012	0.4808	0.010	0.5068
**12-HpETE**	1.79E-04	0.9313	0.143	0.0113	0.001	0.8327	0.002	0.7485	0.005	0.6495	0.057	0.1174	0.016	0.4071	0.037	0.2028	0.000	0.9207
**13-oxoODE**	0.001	0.8083	0.078	0.0670	0.030	0.2505	0.057	0.1179	1.89E-04	0.9294	0.033	0.2404	5.77E-04	0.8770	0.056	0.1176	0.001	0.82
**13-HODE**	0.009	0.5316	0.016	0.4081	0.011	0.4978	0.006	0.6036	1.19E-05	0.9822	0.001	0.8173	0.002	0.7725	0.050	0.1410	0.000	0.9158
**9-oxoODE**	0.019	0.3767	0.003	0.7089	0.004	0.6672	0.123	0.0199	0.017	0.4002	5.27E-06	0.9882	0.004	0.7016	0.002	0.7658	0.004	0.6873
**9-HODE**	0.028	0.2744	0.002	0.7857	0.034	0.2288	0.039	0.1993	4.26E-05	0.9664	0.001	0.8297	0.006	0.6085	0.016	0.4028	0.001	0.8694
**18-HEPE**	1.68E-04	0.9335	6.14E-05	0.9597	0.028	0.2681	0.170	0.0054	0.032	0.2430	0.036	0.2163	0.046	0.1623	0.099	0.0350	0.012	0.4704
**17-HDHA**	0.002	0.7583	0.006	0.6218	0.031	0.2401	0.063	0.1017	4.88E-04	0.8868	0.004	0.7000	0.010	0.5197	0.031	0.2474	0.002	0.7955
**14-HDHA**	0.007	0.5803	0.036	0.2195	0.002	0.7953	9.91E-04	0.8393	7.66E-04	0.8584	0.003	0.7188	0.010	0.5107	0.020	0.3509	0.002	0.7877
**5,12-DiHETE**	0.054	0.1280	0.103	0.0338	0.022	0.3267	0.004	0.6943	0.026	0.3003	0.058	0.1165	0.022	0.3381	0.009	0.5354	0.000	0.9059
**8,9-DiHETE**	0.025	0.3057	0.054	0.1282	2.98E-04	0.9136	0.184	0.0051	0.038	0.2146	0.003	0.7433	0.058	0.1155	0.001	0.8222	0.039	0.2056
**2-AG**	0.020	0.3627	0.069	0.0840	0.251	0.0004	0.312	<0.0001	0.242	0.0007	0.021	0.3531	0.016	0.4163	0.002	0.7730	0.000	0.9679
**AEA**	0.026	0.2958	0.010	0.5098	0.010	0.5039	2.12E-04	0.9253	5.88E-07	0.9961	0.042	0.1847	0.009	0.5413	0.087	0.0495	0.003	0.737
**OEA**	0.100	0.0365	0.008	0.5701	0.033	0.2288	0.072	0.0777	0.043	0.1788	0.078	0.0658	0.033	0.2395	0.022	0.3297	0.014	0.4262
**PEA**	0.047	0.1566	0.017	0.3939	0.006	0.6087	0.184	0.0037	0.077	0.0691	0.104	0.0325	0.005	0.6541	3.11E-04	0.9085	0.008	0.5531
**LA**	0.004	0.6823	0.093	0.0445	0.131	0.0134	0.137	0.0134	2.13E-04	0.9251	0.105	0.0320	0.003	0.7102	0.095	0.0396	0.016	0.3915
**AA**	0.046	0.1622	0.001	0.8082	0.006	0.6164	0.167	0.0058	0.036	0.2187	0.095	0.0421	4.48E-04	0.8916	0.100	0.0342	0.022	0.3213
**EPA**	0.032	0.2449	0.029	0.2699	0.005	0.6510	0.165	0.0062	0.004	0.6918	0.002	0.7511	0.098	0.0381	0.168	0.0052	0.033	0.2246
**DHA**	0.017	0.3967	0.006	0.6262	0.004	0.6894	0.225	0.0011	0.002	0.7635	0.018	0.3907	0.085	0.0552	0.114	0.0235	0.061	0.093

Table A2. Data shown are presented as median (IQR) for the KOOS-4 and Tegner scores of the KICK participants included in this sub-study over study time points.

	**KICK participants (this sub-study)**
No. of Participants	47
KOOS-4 Score	
Baseline	39.7 (27.5-48.1)
3 Months	62.6 (51.4-71.6)
2 Years	82.6 (68.6-90.9)
Tegner Score:	
Pre-injury	8.5 (6-10)
Baseline	2.0 (1-3)
3 Months	4.0 (4-5)
2 Years	6.0 (5-9)
